# Malaria-Related Host Genetic Variation in an Endemic Population of Southern Senegal

**DOI:** 10.3390/ijms27188123

**Published:** 2026-09-12

**Authors:** Babacar Souleymane Sambe, Aissatou Diagne, Jody E. Phelan, Nina Billows, Helene Ataume Mawounge Diatta, Mark K. I. Tan, Serigne Ousmane Mbacké Diaw, Arona Sabène Diatta, Ibrahima Sarr, Inès Vigan-Womas, Leen N. Vanheer, Susana Campino, Taane G. Clark, Makhtar Niang

**Affiliations:** 1 Pole Immunophysiopathology and Infectious Diseases, Institut Pasteur de Dakar, 36 Avenue Pasteur, Dakar 220, Senegal; babacarsouleymane.sambe@pasteur.sn (B.S.S.); ousmanembacke.diaw@pasteur.sn (S.O.M.D.);; 2 Faculty of Infectious and Tropical Diseases, London School of Hygiene and Tropical Medicine, London WC1E 7HT, UKleen.vanheer@lshtm.ac.uk (L.N.V.);

**Keywords:** malaria, host genetic variation, balancing selection, Senegal

## Abstract

Host genetic variation is a key determinant of malaria susceptibility, particularly in endemic regions where intense transmission has driven strong selective pressures on red blood cell-related genes. However, Senegalese and other African populations remain underrepresented in genomic studies, limiting population-specific inference. We analyzed malaria-related host genetic variants in 140 febrile patients from southern and south-eastern Senegal, focusing on key loci (ACKR1, Dantu blood group, HBB and G6PD) and on composite Multilocus Genotype Profiles (MGPs). We described the co-occurrence of the variants within individuals, allowing exploratory assessment of combined genetic architectures. We identified both well-established malaria-protective variants, including the Duffy-negative Fy(a–b–) phenotype (observed in all individuals), G6PD key variants (e.g., G202A with an allelic frequency of ~3%, A376G at ~40%, T968C at ~1%), and HBB variants (HbS at ~7% and HbC at ~1%), as well as some rare and less characterized polymorphisms. To the best of our knowledge, this study provides the first description in the Senegalese population of the Dantu variant (rs186873296) and the rare ACKR1 rs577808287 variant, each observed in 2 distinct individuals. Five major composite MGPs across the four loci were predominantly observed and together represented more than 75% of individuals. Excluding Duffy status, most of these profiles included at least one variant previously reported in the literature as malaria-protective, a pattern consistent with the hypothesis of malaria-driven balancing selection, despite the presence of potentially deleterious alleles. This study highlights substantial genetic heterogeneity in malaria-relevant host genes in Senegal and demonstrates the value of integrating composite genetic architectures into malaria research. These findings provide population-specific data that are critical for improving malaria risk assessment, clinical interpretation, and public health strategies in endemic regions. Moreover, the relatively high frequencies of some variants may reflect balancing selection, possibly partially driven by the adaptive benefits they confer in populations exposed to malaria.

## 1. Introduction

Malaria is a life-threatening disease caused by *Plasmodium* parasites and transmitted to humans through the bites of infected mosquitoes. In 2024 alone, it accounted for an estimated 282 million cases and 610,000 deaths worldwide, with children in sub-Saharan Africa bearing a disproportionately large share of the burden [1]. Malaria has been one of the strongest selective forces acting on the human genome, particularly in sub-Saharan Africa, where transmission has remained intense over long evolutionary timescales [2,3]. Human genetic variation plays a major role in shaping susceptibility to infection, disease severity, and clinical outcomes, with host genetic factors accounting for approximately one quarter of the variability in malaria incidence and severity [4]. This selective pressure has favored the emergence and maintenance of multiple protective genetic variants, most of which affect red blood cell biology and host–parasite interactions.

Classical malaria-associated adaptations include hemoglobinopathies, the sickle cell polymorphism (HbS), Glucose-6-Phosphate Dehydrogenase (G6PD) deficiency (e.g., A^−^), and major erythrocyte blood group polymorphisms, such as the Duffy-negative phenotype [3]. These variants illustrate a paradigm of balancing selection, whereby alleles that confer protection against severe malaria are maintained in populations despite potentially deleterious effects in specific genetic or clinical contexts [2,5]. More recently, improved genomic approaches have enabled the identification of additional protective variants, including the Dantu blood group variant and polymorphisms in genes involved in erythrocyte membrane dynamics and immune regulation [3,6]. However, together these known variants explain only a fraction of the heritable component of malaria susceptibility, underscoring its complex and polygenic architecture [4,7].

Despite extensive evidence for the role of host genetics in malaria, genomic studies remain strongly biased toward populations of European ancestry, with African populations—who harbour one of the greatest human genetic diversity—being markedly underrepresented [8]. Within Africa, most host genetic studies of malaria have been conducted in East Africa, while West Africa, and Senegal in particular, remain comparatively underexplored [7,9]. This limited representation constrains population-specific interpretation of malaria-associated variants and hampers the identification of locally relevant genetic combinations shaped by distinct epidemiological and evolutionary contexts, particularly because malaria susceptibility is likely influenced not only by individual variants but also by the combined effect of multiple co-occurring host genetic factors.

The lack of detailed host genetic data has important clinical and public health implications. Several malaria-protective variants influence not only disease susceptibility but also treatment safety and drug response. G6PD deficiency, for example, confers partial protection against severe malaria but increases the risk of hemolysis following exposure to certain antimalarial drugs or oxidative stressors [10,11], highlighting the relevance of pharmacogenetics in endemic settings. Accounting for population-specific genetic diversity is therefore essential for optimizing malaria case management, drug policy, and elimination strategies.

In Senegal, malaria remains a major public health burden, with persistent transmission of *Plasmodium falciparum* and increasing recognition of non-falciparum species, particularly in eastern and southern regions [12,13,14,15]. While epidemiological and parasitological surveillance has improved, the contribution of human genetic diversity to malaria susceptibility, clinical presentation, and treatment response is rarely integrated into control strategies. Existing studies have documented the presence of key HBB (Hemoglobin subunit beta) and G6PD variants and their associations with malaria severity and hematological parameters, but these investigations have generally focused on individual polymorphisms rather than their combined genetic context [9]. Exploring the co-occurrence of malaria-related variants may therefore provide additional insight into the composite genetic architectures shaped by long-term malaria exposure in endemic populations.

In this study, we investigate malaria-relevant host genetic variation in febrile patients from southern and south-eastern Senegal, a region characterized by high malaria transmission and circulation of multiple *Plasmodium* species [12]. By analyzing key genes involved in red blood cell biology, including ACKR1 (Atypical Chemokine Receptor 1), Dantu blood group, G6PD, and HBB, and by examining both individual variants and their co-occurrence as composite Multilocus Genotype Profiles (MGPs), used here as an exploratory framework to describe combined malaria-related genetic architectures within individuals, we aim to generate population-specific genetic data from an underrepresented setting and to explore how composite genetic architectures reflect malaria-driven balancing selection. This work seeks to contribute to a more refined understanding of host–malaria interactions in Senegal and to support the integration of human genetic diversity into malaria research, clinical practice, and control strategies.

## 2. Results

### 2.1. Study Population Demographics

This study used a targeted long-read sequencing approach to characterize malaria-related host genetic variants in 140 febrile patients recruited in southern and south-eastern Senegal between 2020 and 2022 with four malaria-related loci. Demographics of the study participants summarized in Table 1 indicated that 25.71% of patients were sampled in 2020, 44.29% in 2021, and 30% in 2022. Among the 140 samples, 22.14% (31/140) originated from Kédougou, 61.43% (86/140) from Tambacounda, and approximately 16.42% from the southern regions of Kolda and Ziguinchor, which were grouped into a single geographic category due to the limited sample sizes. The gender distribution was balanced, with a male-to-female ratio close to one (69/70 = 0.99). Age stratification indicated a predominantly young population, with more than 55% of participants under 20 years of age (born in the 2000s), approximately 32% aged between 20 and 44 years (born between the 1980s and 2000s), and only 10% were aged 45 years or older (born before the 1980s).

### 2.2. Genetic Polymorphism and Diversity

**Figure 1:**
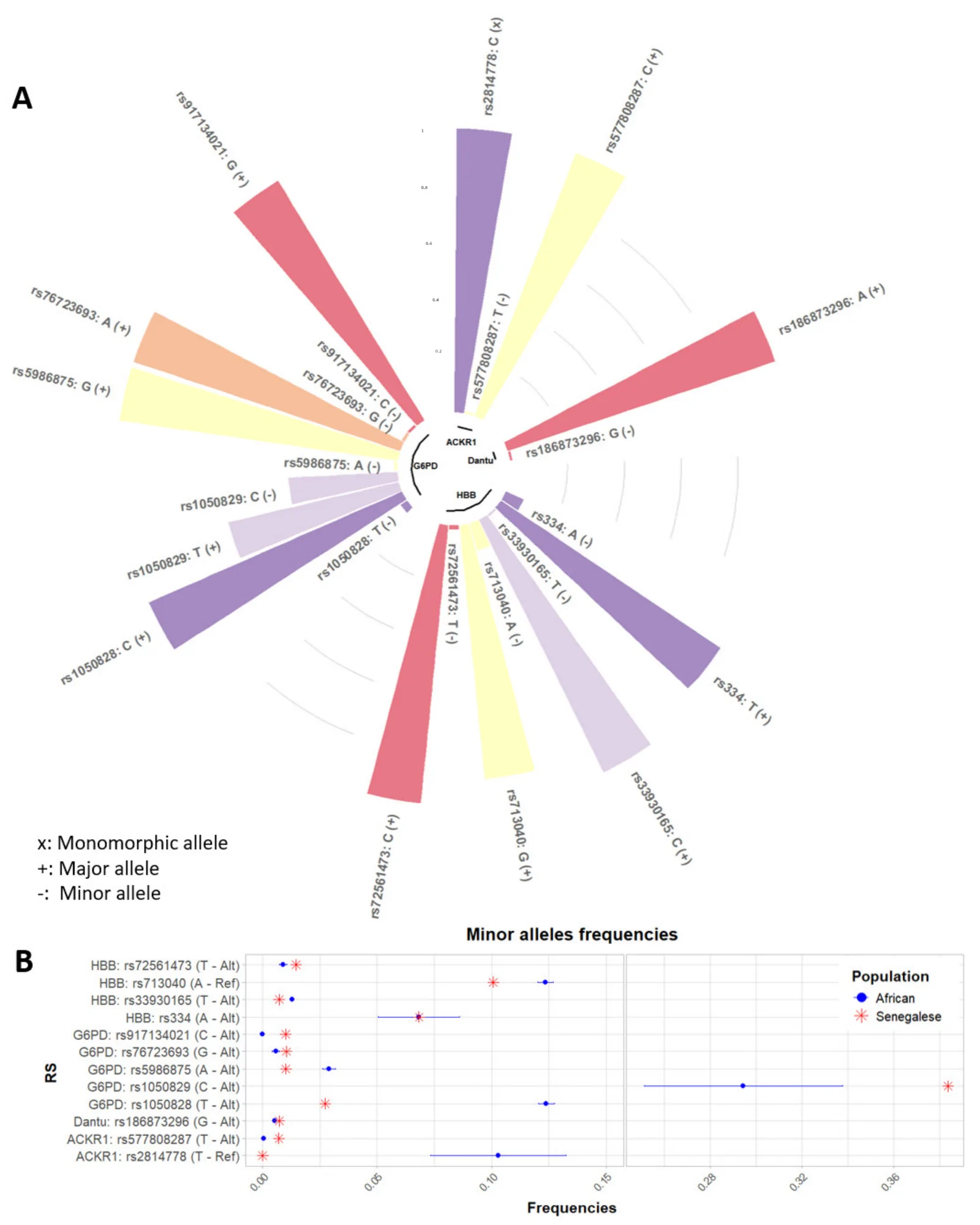
Frequency of G6PD, ACKR1, HBB and Dantu alleles in Senegalese population (**A**) compared to mean African population (**B**).

A total of 12 genetic variants were identified across the four analyzed loci (Figure 1; Supplementary Tables S1 and S2). These comprised five variants in G6PD, including G202A (rs1050828), A376G (rs1050829), T968C (rs76723693), as well as rs5986875 and rs917134021, four variants in HBB, namely HbS (rs334), HbC (rs33930165), rs713040, a variant associated with β-thalassemia, and rs72561473- two variants in ACKR1 (rs2814778, corresponding to the Fy(a–b–) phenotype, and rs577808287), and the Dantu variant (rs186873296).

Overall genetic diversity within the study population was low (heterozygosity ≈ 5%), with no evidence of genetic differentiation between geographic zones, as indicated by near-zero Fst values (Supplementary Table S3). In addition, no significant linkage disequilibrium was detected between analyzed loci (Supplementary Figure S1).

Among these, ACKR1 rs2814778 was monomorphic, with all individuals carrying the Fy(a–b–) genotype. All other loci were polymorphic, although most variants were observed at low frequencies (<5%). Notable exceptions included G6PD A376G (rs1050829), which showed an allele frequency of approximately 40%, HBB rs713040 detected at a frequency of approximately 10%, and HbS (rs334) with an allele frequency close to 7% (Figure 1A,B). Remarkably, one person had sickle cell disease (HbS, SS genotype).

### 2.3. Allelic and Genotypic Frequencies of Medically Relevant Variants and Comparison with African Reference Populations

**Figure 2:**
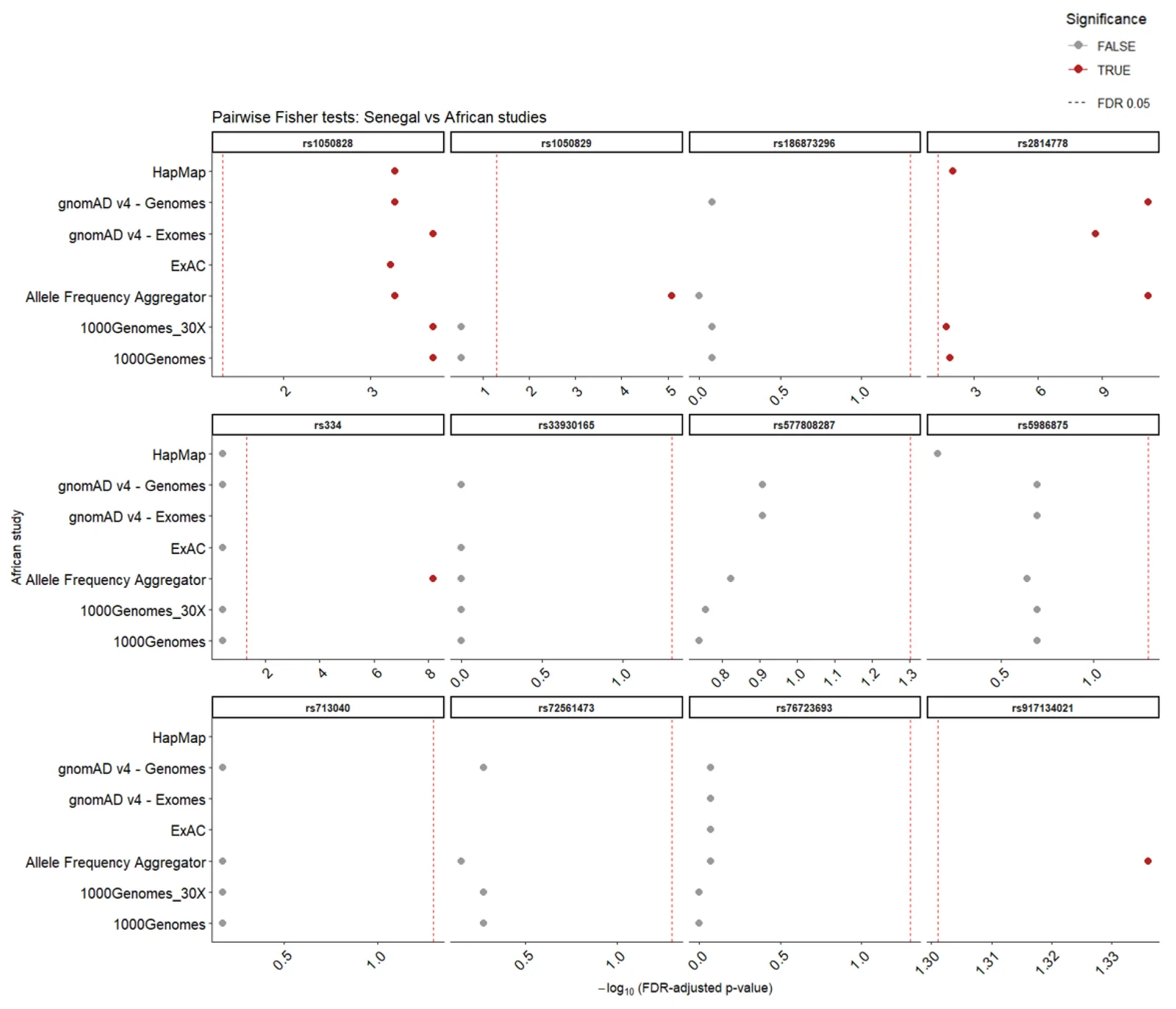
Pairwise comparison of allele frequencies between Senegal and African studies.

Comparison of Senegalese allele frequencies with aggregated African reference populations revealed substantial differences for several loci (Figure 1B). The G6PD rs1050829 (A376G) allele was more frequent in Senegal (0.384) than in African reference populations (0.294 ± 0.043), and the rs917134021 variant was detected at a low frequency in Senegal (<0.05) but was absent from the African reference datasets. By contrast, the G6PD rs1050828 (G202A) allele was less frequent in Senegal (0.027) compared to African reference estimates (0.124 ± 0.003). These differences were statistically significant (Figure 2), indicating that allele frequency distributions at several medically relevant loci in Senegal deviate from those reported in large-scale African genomic studies.

### 2.4. Spatial and Age-Stratified Distribution of Variants

**Figure 3:**
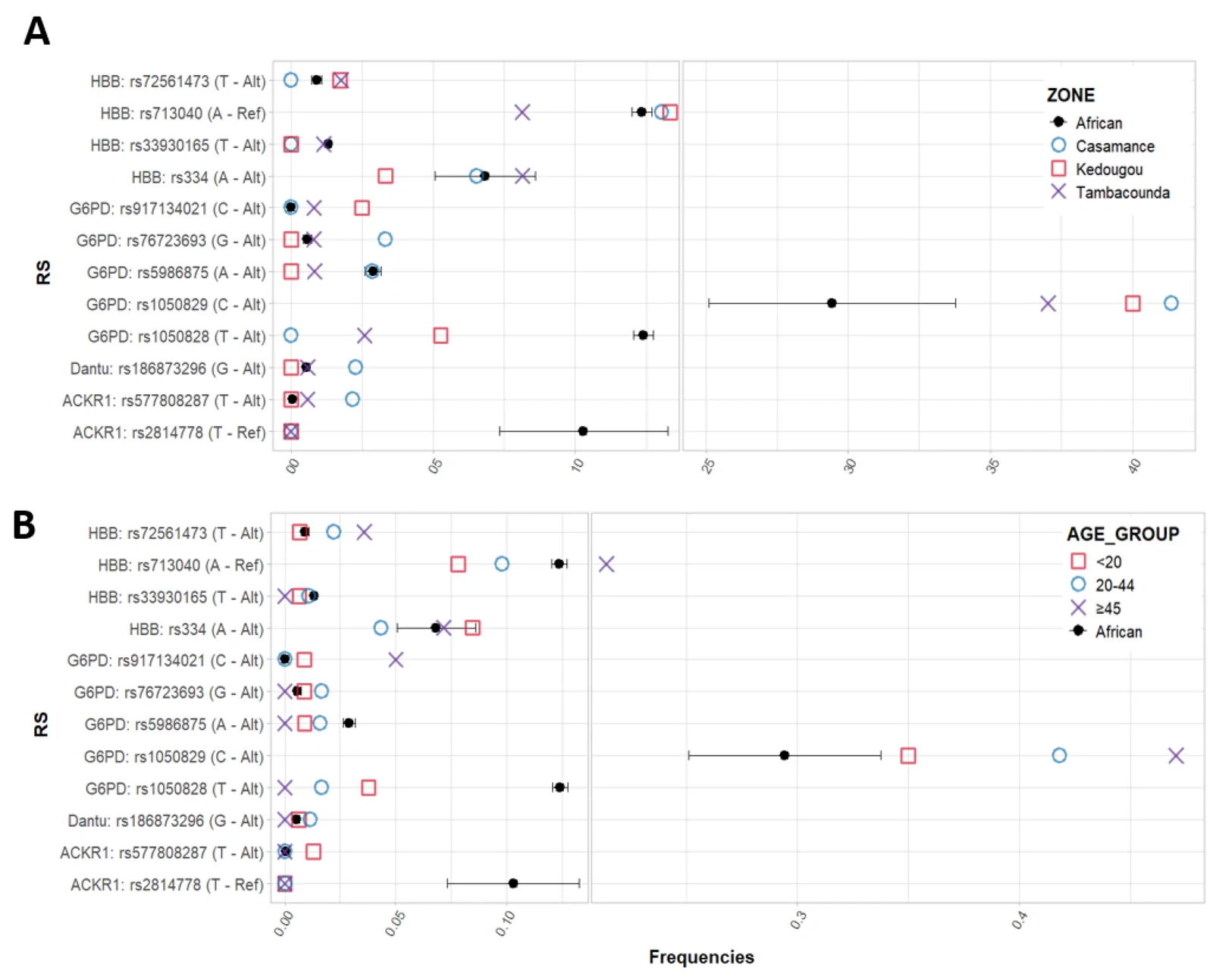
Allele frequency comparisons across populations, regions (**A**), and age groups (**B**).

Analysis of variant distribution across study sites revealed geographically restricted occurrences for several rare variants (Figure 3A). For instance, HBB rs72561473 (mutation associated with β-thalassemia) was detected exclusively in the south-eastern zone, with four individuals carrying the variant (3 in Tambacounda and 1 in Kédougou). The Dantu variant (rs186873296) and ACKR1 rs577808287 (mutations that potentially affect the gene regulation) were each observed only in 2 samples from Ziguinchor.

Age-stratified analysis further highlighted differences in allele frequencies across age groups (Figure 3B). Notably, the frequencies of HBB rs72561473 and G6PD rs1050829 decreased across age groups, whereas G6PD rs1050828 exhibited a progressive increase. These patterns suggest age-dependent differences in the allele frequencies.

### 2.5. Distribution of Multilocus Genotype Profiles (MPGs)

G6PD haplotype-based analysis (Table 2) revealed that in males (hemizygotes), the B variant frequency was approximately 73.5%, while the A variant frequency was 26.5%. In females, the frequencies were 57.3% for B, 37.1% for A (35.5% + 1.6%), and 5.6% (2.6% + 1.6% + 1.4%) for the A^−^ variant (associated with a high risk of hemolytic anemia).

**Figure 4:**
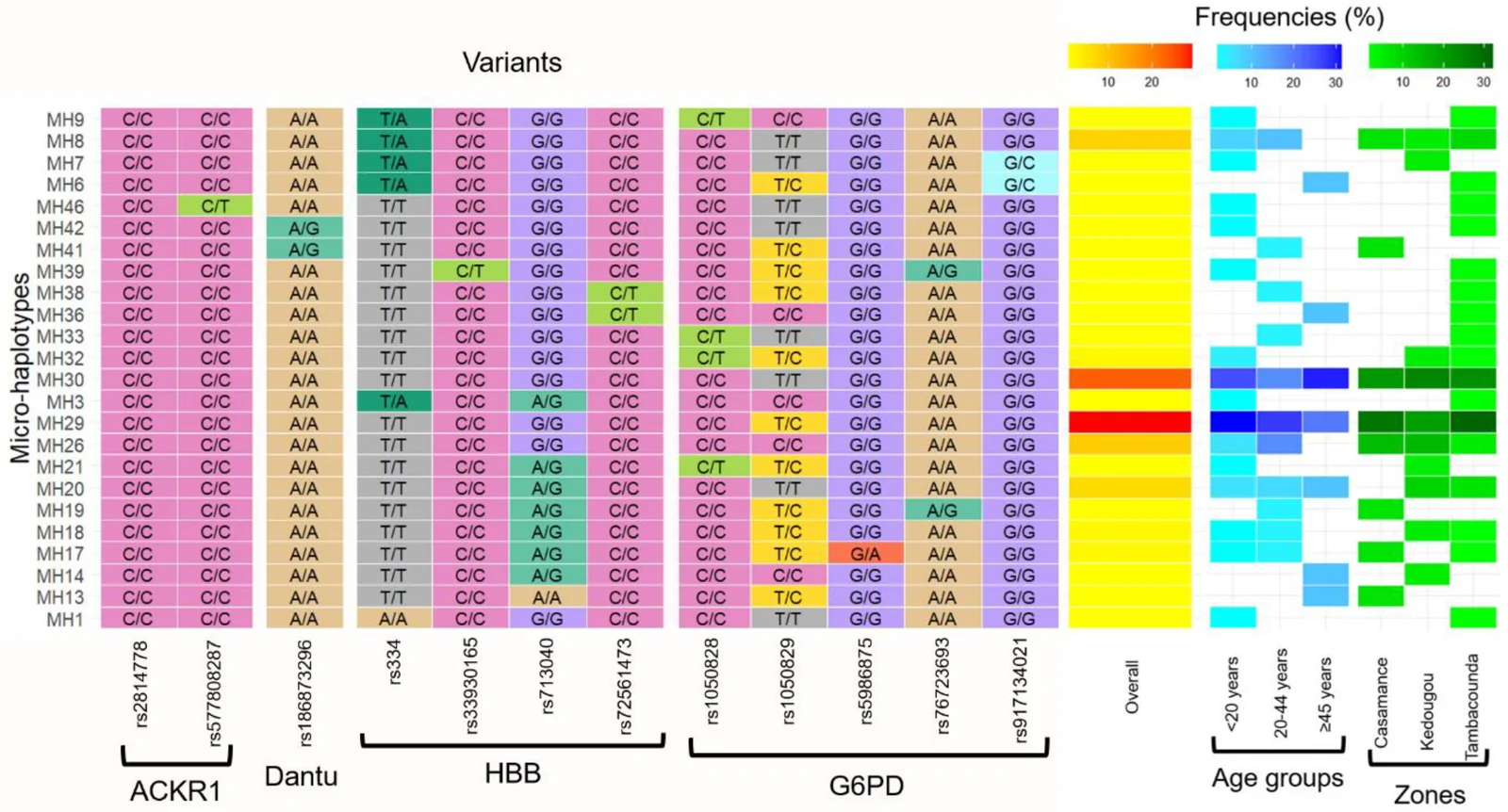
Age-stratified and spatial patterns of MPGs distribution (only individuals with complete data were considered for this analysis; males were considered homozygous for G6PD).

Across the study population, there were 24 characterised MPGs (with complete genotypic data), with five accounting for more than 75% of all observed combinations (Figure 4, Supplementary Figure S2). These included MH29 (~29%), MH30 (~24%), MH8 (~9%), MH20 (~8%), and MH26 (~7%). MH29 and MH30 were detected across all age groups and geographic zones, whereas MH20 was absent in the Southern zone, and MH08 and MH26 were not observed in individuals aged ≥45 years.

Considering only variation at all loci excluding the rs2814778 in ACKR1 (since all individuals were Duffy-negative [Fy(a–b–)]), these five most frequent MPGs were characterized by distinct combinations of variants at the HBB and G6PD loci (Table 3). MH29 was defined by individuals homozygous (GG) at rs713040 combined and heterozygous for G6PD rs1050829 (A376G), whereas MH30 comprised homozygous individuals (GG) at rs713040 without additional variants at the analyzed loci. MH8 was characterized by individuals homozygous (GG) at rs713040 in combination with the HbA/HbS (rs334, AS) genotypes. MH20 included individual heterozygous (AG) at rs713040, while MH26 corresponded to individuals homozygous (GG) at rs713040 together with homozygosity for G6PD rs1050829 (A376G).

## 3. Discussion

This study enabled the detection of multiple clinically relevant host genetic variants previously associated in the literature with malaria susceptibility and resistance, including variants that are potentially reported for the first time in Senegal and possibly in West Africa. The Dantu variant (rs186873296, A>G) was identified in two heterozygous individuals (~1%) from Ziguinchor in southern Senegal. To our knowledge, this represents the first description of this variant in Senegal. Dantu has previously been shown to confer strong protection against severe *P. falciparum* malaria by modifying red blood cell biomechanics and limiting parasite invasion [16,17]. Although its frequency can reach up to 9.5% in Kenyan populations, it is generally absent or extremely rare outside Eastern Africa [18,19]. Its detection in our cohort underscores the pronounced spatial heterogeneity of malaria-resistance variants across Africa and suggests that variants previously considered to be geographically restricted in East Africa may occur at very low frequencies in West African populations, yet remain poorly documented due to limited local genomic studies.

Beyond this novel finding, our study also identified several well-characterized in the literature malaria-associated variants with established clinical relevance. At the ACKR1 locus, all individuals carried the Fy(a–b–) phenotype, which is nearly fixed in sub-Saharan Africa and has been linked to resistance to *P. vivax* infection by preventing parasite entry into red blood cells [3]. This observation is consistent with long-standing evidence of strong malaria-driven selection acting on this locus. Similarly, at the G6PD gene, we detected three classical coding variants—G202A, A376G, and T968C—that are extensively documented across Africa and form the core of the common African G6PD A^−^ haplotypes. These variants are associated with reduced enzyme activity and an increased risk of hemolytic anemia under oxidative stress, including infections and exposure to certain antimalarial or oxidative drugs [20,21,22]. In line with previous studies from Senegal, our results confirm marked regional and gender-specific heterogeneity in G6PD variation, with deficient A^−^ haplotypes coexisting alongside a high prevalence of the G6PD A genetic background, particularly among women.

In addition, several HBB variants with well-established links to malaria were identified, including HbS (~7%) and HbC (~1%). HbS is one of the most extensively studied human polymorphisms in Africa and is maintained by malaria-driven balancing selection: while homozygosity causes sickle cell disease, observed in one individual, and heterozygous carriers benefit from substantial protection against severe malaria [5,23,24]. HbC, which is largely restricted to West Africa, has also been associated with protection against severe malaria, particularly in homozygous individuals, although its effect appears more variable and population-dependent [25,26,27]. The frequencies observed in our cohort are consistent with previous reports from Senegal and neighboring countries, reinforcing the central role of HBB variation in shaping malaria susceptibility at the population level.

Alongside these well-characterized variants, our study also identified some less well-documented polymorphisms whose functional and clinical relevance remain uncertain. These include the rare variant ACKR1 rs577808287, two intronic variants in G6PD (rs5986875 and rs917134021), and two HBB variants (rs713040 and rs72561473). As an example, for rs917134021, available information remains scarce, as allele frequency data currently available in dbSNP (NCBI) are derived from the ALFA project in which individuals of European ancestry account for approximately 76.4% (14,148/18,512) of the genotyped samples, with other global populations contributing only a minor proportion [28]. Current evidence suggests that these variants are largely benign and have no clearly established impact on enzyme activity, malaria susceptibility, or clinical outcomes. While the HBB rs713040 variant has not yet been clearly associated with protection against malaria, it is known to be linked to benign forms of β-thalassemia and to hereditary persistence of feotal hemoglobin (HPFH). Notably, β-thalassemia-related phenotypes have been widely described as conferring protection against malaria [3,6,29]. Similarly, the intronic G6PD variants and the rare upstream HBB rs72561473 variant remain poorly characterized and currently lack evidence of functional or clinical significance [28,30]. The detection of these low-frequency and under-annotated polymorphisms highlights the substantial genetic diversity present among Senegalese populations and emphasizes the importance of locally generated genomic data to refine variant annotation and clinical interpretation in malaria-endemic settings.

Beyond the analysis of individual variants, our study provides an integrated view of how malaria-relevant mutations co-occur within individuals living in a high-transmission environment. Aside from the universal Duffy-negative phenotype, the major MPGs identified in our cohort (MH29, MH30, MH8, MH20, and MH26), together accounting for approximately 76% of individuals, all carry at least one variant with a documented or supposed protective effect against malaria, including benign forms of β-thalassemia, the HbA/HbS genotype, or the G6PD A376G variant. These composite genetic profiles combine variants whose protective roles against severe *P. falciparum* malaria, particularly in heterozygous carriers, are well-established [5,23,24,25,26]. Importantly, although some of these variants are traditionally associated with potentially deleterious clinical effects, such as G6PD deficiency or mild hematological changes, their adverse impact is often attenuated in endemic settings by genetic background, environmental exposure, and clinical context. Under intense malaria pressure, the survival advantage conferred by these variants may substantially outweigh their biological costs, which are frequently mild, context-dependent, or clinically silent in the absence of specific triggers [4,6,21,22,31]. The high prevalence and persistence of these composite genetic architectures are therefore consistent with patterns expected under balancing selection, potentially reflecting an evolutionary trade-off between infectious pressure, host adaptation, and clinical risk.

Some limitations should be considered for this study. It is primarily a descriptive and exploratory characterization of malaria-related host genetic variants in a Senegalese population living in malaria endemic regions rather than a direct genetic association study. The overall sample size and the number of individuals per geographic zone were modest, particularly in southern regions, which have limited the power to detect subtle differences in allele frequencies or genetic diversity across regions or age groups. Consequently, the lack of strong genetic differentiation should be interpreted cautiously within the spatial and temporal scope of the study. Additionally, the study population consisted of febrile patients attending health facilities, which may not fully reflect the genetic composition of the general population, and therefore allele frequency estimates should not be interpreted as representative of population-level prevalence. Furthermore, while variant calling followed stringent quality filtering procedures appropriate for high-throughput amplicon sequencing, the study was not designed to include routine orthogonal validation (e.g., Sanger sequencing or ddPCR), which may be considered in future large-scale or confirmatory studies to further support rare variant characterization. Moreover, we did not assess X-chromosome inactivation (lyonization) in heterozygous females, which produces a mosaic of G6PD-normal and -deficient red blood cells. The ratio of the two cell types is highly variable, and cannot be predicted from genotype alone [20,32]. Nevertheless, this recruitment strategy remains epidemiologically relevant in malaria-endemic settings, as it captures individuals most likely to have experienced malaria-related selective pressures. In addition, no systematic information on ancestry or country of origin was collected at enrolment; however, southern and south-eastern Senegal are highly mobile and genetically admixed regions with substantial population mixing across West Africa, which may introduce additional unmeasured genetic heterogeneity. Interpretation is further constrained by the underrepresentation of African populations in global genomic databases, where individuals of African ancestry account for less than 2% of participants despite harboring the greatest human genetic diversity [8,33]. Despite these limitations, this study provides timely insights into the genetic landscape of malaria-related human variants in southern and south-eastern Senegal, a region with a high malaria burden and co-circulation of *P. falciparum* and non-falciparum species [12]. In a context where substantial technological and infrastructural barriers limit large-scale human genomics research in Africa [34,35], even moderately sized, well-characterized datasets contribute meaningfully to filling critical knowledge gaps and improving the representation of African populations in human genetic research [8,33].

## 4. Materials and Methods

### 4.1. Study Area

**Figure 5:**
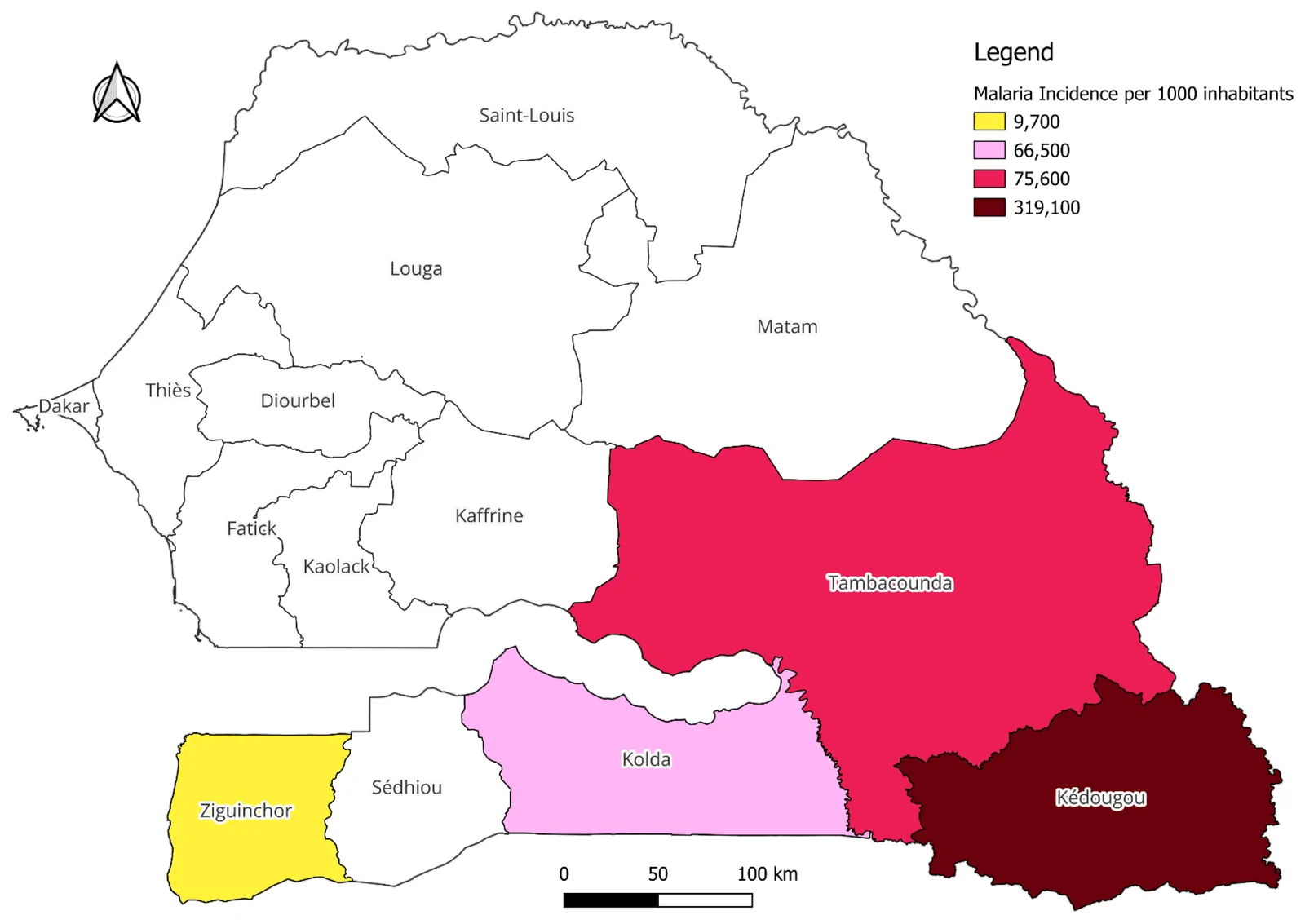
Map of Senegal depicting the heterogeneity of malaria incidence across the country in 2024 [36] and the regions in the study area in the South and Southeastern areas of the country.

The study was conducted in the southern and south-eastern regions of Senegal (Figure 5), areas characterized by intense and heterogeneous malaria transmission. According to national surveillance data [36], Senegal reported 429,369 confirmed malaria cases in 2024, representing an increase of more than 21% compared to 2023. Among these cases, 10,433 were classified as severe malaria (+11% compared to 2023), with 314 reported deaths and a national incidence of 22.8‰ [36].

The regions included in this study were Kédougou and Tambacounda, located in the south-eastern part of the country, and Kolda and Ziguinchor, located in southern Senegal. Despite their relatively low population density (15% of Senegal’s population) [37], these four regions bear a disproportionate share of the malaria burden [36]. In 2024, they collectively accounted for approximately 215,464 malaria cases, corresponding to more than 50% of the nationwide reported cases. Specifically, Kédougou, Tambacounda, Kolda, and Ziguinchor accounted respectively for 16.2%, 17.8%, 14.5%, and 1.8% of national malaria cases, respectively, and together represented 15.4% of severe malaria cases nationwide.

Marked differences were also observed in malaria lethality rates. Compared to the 3% national average in 2024, lethality in three of the four study regions ranged from 1.5 to 2.6 times higher, whereas Ziguinchor showed a lower rate (0.6 times the national rate).

### 4.2. Study Population and Sampling

This study was conducted within the framework of a malaria molecular surveillance program among febrile patients in southern and south-eastern Senegal [12]. Data and biological samples were collected between 2020 and 2022 among eligible participants aged six months or older with an axillary temperature greater than 37.5 °C and at least one symptom compatible with malaria such as headache, nausea, dizziness, chills, or fatigue.

Written informed consent was obtained from all adult participants, while assent and consent from parents or guardians were obtained for minors prior to enrolment. Venous blood samples were collected in EDTA tubes for molecular analyses. Demographic information, including age and sex, was recorded in a dedicated registry for analysis purposes.

### 4.3. DNA Extraction, Gene Amplification and Sequencing

Genomic DNA (gDNA) was extracted from approximately 200 µL of frozen whole blood using the QIAamp DNA Blood Mini Kit (Qiagen, Hilden, Germany), following the manufacturer’s instructions. Extracted DNA was eluted in 100 µL nuclease-free water and stored at −20 °C until further use.

Four human genes associated with malaria susceptibility and disease severity were targeted for amplification: HBB (Hemoglobin subunit beta; chromosome 11), ACKR1 (Atypical Chemokine Receptor 1, formerly DARC; chromosome 1), G6PD (Glucose-6-Phosphate Dehydrogenase; X chromosome), and the Dantu blood group locus (glycophorin (GYP) gene cluster; chromosome 4). Primer sets were designed to amplify genomic regions encompassing medically relevant polymorphisms, generating amplicons ranging from approximately 400 to 600 base pairs. The targeted variants included, among others, HbS (rs334), HbC (rs33930165) and regulatory HBB variants, the Duffy-null variant in ACKR1 (rs2814778), multiple functional and regulatory variants in G6PD (including rs1050828, rs1050829, rs76723693), and the Dantu variant (rs186873296). Detailed primer sequences and amplification conditions were previously described by Osborne et al. and are available on the LSHTM PathogenSeqLab—Human genomic GitHub repository [6,38].

Amplified products were purified and pooled before sequencing. Libraries were prepared following the Oxford Nanopore Technologies ligation sequencing amplicon protocol using the Native Barcoding Kit 96 (SQK-NBD114.96, v14), according to the manufacturer’s instructions, and sequenced on ONT platforms (MinION Mk1B, R10.4.1 flow cells FLO-MIN114) for 24h. Nanopore sequencing was performed to enable long-read, high-throughput characterisation of targeted genomic regions. Basecalling using Dorado (version 0.7.3), demultiplexing, and initial quality control were conducted using ONT standard bioinformatics pipelines.

Raw sequencing reads generated using ONT were basecalled, demultiplexed, and quality-filtered using standard ONT pipelines. Reads were aligned to the human reference genome (GRCh38), and variant calling was restricted to predefined target regions corresponding to the amplified loci. Variants were called using FreeBayes with a minimum coverage threshold of 30x and a minimum base quality score of 30, followed by normalization and filtering using bcftools. Only variants passing quality filters (FMT/DP > 30 and QUAL > 30) were retained for downstream analyses. Functional annotation was performed using bcftools csq and SnpSift against ClinVar reference databases. Bioinformatics scripts, from demultiplexing to downstream data filtering, are also available on the LSHTM PathogenSeqLab—Human genomic GitHub repository [38].

### 4.4. Data Analysis

Allele and genotype frequencies were estimated for all detected variants in the overall study population and further stratified by geographical zone and age group where relevant. For X-linked G6PD variants, allele and genotype frequencies were calculated separately for males and females to account for sex-specific inheritance patterns.

Genetic diversity was characterized using observed heterozygosity (H_O_), expected heterozygosity (H_E_), and the inbreeding coefficient (F_IS_), while genetic differentiation between geographical zones was assessed using fixation indices (F_ST_). Together, these parameters were used to describe within-population diversity and to explore potential population structure across the study area. Pairwise linkage disequilibrium (LD) between polymorphic loci, calculated using R^2^ and Fisher’s exact test, was also evaluated to identify non-random associations among variants, with statistical significance assessed after correction for multiple testing.

Allele frequencies observed in the Senegalese population were compared with those reported for aggregated African reference populations. Reference data were retrieved from the National Center for Biotechnology Information (NCBI) [39] and derived from large-scale genomic resources, including the Genome Aggregation Database (gnomAD; exomes and genomes) [40], the Allele Frequency Aggregator (ALFA) [41], the 1000 Genomes Project (standard and 30× datasets) [42], the International HapMap Project [43], and the Exome Aggregation Consortium (ExAC) [44]. Statistical comparisons were performed for variants with sufficient data, and p-values were adjusted using the Benjamini–Hochberg false discovery rate (FDR) procedure.

Spatial analyses were conducted by comparing allele frequencies across predefined geographical zones, whereas age-stratified analyses were used as a proxy for temporal trends. These analyses were intended to identify patterns in variant distribution rather than to infer strong population differentiation.

MPGs (co-occurrence of genotypes) were reconstructed by combining variants across the HBB, ACKR1, G6PD, and Dantu blood group loci. MPGs frequencies were estimated for the overall population and stratified by geographical zone and age group. For G6PD, a microhaplotype-based approach was employed to infer genotypic profiles associated with normal or reduced enzymatic activity [45,46].

Analyses were restricted to individuals with available genotypic and demographic data for the variables under consideration. Individuals with incomplete multilocus genotypes were excluded from composite profile reconstruction analyses. All statistical analyses were performed using R (version 4.3.2) [47], and figures and summary statistics were generated using appropriate population genetics and data visualization packages.

## 5. Conclusions

Focusing on febrile patients, this study targets a population of direct epidemiological relevance in malaria-endemic settings, that is likely to undergo malaria diagnosis, treatment, and antimalarial drug exposure. Consequently, the observed frequencies and combinations of clinically relevant host genetic variants, particularly those affecting red blood cell biology and drug tolerance, such as G6PD deficiency-related variants, have immediate implications for public health and clinical decision-making.

Beyond the analysis of individual variants, this work documents the presence, distribution, and co-occurrence of multiple malaria-associated variants previously described in the literature in southern and south-eastern Senegal, regions that remain underrepresented in large-scale African genomic reference datasets. Importantly, we identified both well-established protective variants and less well-documented polymorphisms, including the Dantu variant (rs186873296), a very rare ACKR1 variant (rs577808287), and some HBB and G6PD mutations whose functional and clinical consequences remain poorly characterized. Together, these findings provide population-specific genetic data and highlight the substantial genetic heterogeneity shaping malaria susceptibility in this setting.

Finally, these results lay the foundation for future population-based, longitudinal, and pharmacogenetic studies and emphasize the need to integrate host genetic diversity into malaria control strategies, risk assessment, and antimalarial drug policy planning in endemic regions.

## Figures and Tables

**Table 1:** Study population characteristics.

	South-Eastern Senegal		South Senegal		Overall (n = 140)	Percentage (%)
	Kedougou (n = 31)	Tambacounda (n = 86)	Kolda (n = 15)	Ziguinchor (n = 8)		
**Gender**						
Female	13	44	7	5	69	49.29
Male	18	41	8	3	70	50
NA’s	0	1	0	0	1	0.71
Year						
2020	10	25	1	0	36	25.71
2021	18	41	1	2	62	44.29
2022	3	20	13	6	42	30
Age (years)						
Mean	22.26	19.18	30.27	17.86	21.01	-
Min	2	0.5	8	6	0.5	-
Max	70	82	75	28	82	-
SE	3.11	2.02	4.54	2.78	1.53	-
Age groups (years)						
<20 (born in the 2000s)	18	53	4	3	78	55.71
20–44 (born between the 1980s and 2000s)	9	25	8	4	46	32.86
≥45 (born before the 1980s)	4	7	3	0	14	10
NA’s	0	1	0	1	2	1.43

NA’s: Not available; Min: Minimum; Max: Maximum; SE: Standard Error; -: Not applicable.

**Table 2:** Haplotype-based inference of G6PD genotypes from key SNPs.

Gender	Key SNPs				Frequencies	Inferred G6PD Haplotype
	rs76723693 (T968C)	rs5986875 (G->A)	rs1050829 (A376G)	rs1050828 (G202A)		
Female (2N = 138)	A	G	T	C	0.573	B
	A	G	C	C	0.355	A
	A	G	C	T	0.026	A^−^
	A	A	C	C	0.016	A
	G	G	C	C	0.016	A^−^
	A	G	T	T	0.014	A^−^
Male (N = 70)	A	G	T	C	0.735	B
	A	G	C	C	0.265	A

B: Wild-type, normal activity (100%); A: Near-normal (>60%), usually asymptomatic; A^−^: Deficient (<45%), risk of hemolytic anaemia, variant clinically important or relevant, requiring monitoring and careful drug management.

**Table 3:** Distribution of the most frequent malaria-relevant MPGs and associated clinical variants.

MPGs	Frequency (%)	Relevant Clinical Variants Carried *
MH29	28.89	HBB: rs713040 (GG) + G6PD T376C (heterozygous)
MH30	23.33	HBB: rs713040 (GG)
MH8	8.89	HBB: rs713040 (GG) + HbA/HbS
MH20	7.78	HBB: rs713040 (AG)
MH26	6.69	HBB: rs713040 (GG) + G6PD T376C (homozygous)

* All individuals in this study are Duffy-negative individuals. Only individuals with complete data were considered for this analysis.

## Data Availability

The data supporting the conclusions of this article are included within the article. Additional data may be made available by the authors upon reasonable request.

## Supplementary Materials

The following supporting information can be downloaded at: https://www.mdpi.com/article/10.3390/ijms27188123/s1.
