# Supplementary material for: Malaria-Related Host Genetic Variation in an Endemic Population of Southern Senegal
Source: Int J Mol Sci. 2026 Sep 12;27(18):8123. doi: 10.3390/ijms27188123 (PMC13607421; doi:10.3390/ijms27188123)
Supplement: Supplementary file 1 [file ijms-27-08123-s001.zip › ijms-4319389-supplementary.pdf]

Supplementary Table S1: Genotypic frequencies and description of the impact of detected autosomal variants in the Senegalese population

Chr*	Genes	RS number	Variant information	Genotypes	Observed counts	Total	Frequencies (%)	Consequences / Impacts**
**1**	**ACKR1**	**rs2814778**	Fy(a-b-) T>C	CC	140	140	100	A common variant in Africa (Fy(a-b-)), which confers protection against P. vivax due to the absence of the Duffy receptor on red blood cells
		**rs577808287**	C>T	CC	138	140	98.57	Normal
				CT	2		1.43	A very rare variant located upstream of the gene (~2 kb), which could potentially affect gene expression regulation
**4**	**Dantu**	**rs186873296**	Intergenic A>G	AA	137	139	98.56	non-Dantu
				AG	2		1.44	Dantu heterozygous – rare protective variant against severe P. falciparum malaria and bacterial infections
**11**	**HBB**	**rs334**	HbS	TT	121	139	87.05	HbA/HbA
				TA	17		12.23	Missense coding variant - pathogenic variant causing sickle cell disease, but protects against severe malaria in heterozygotes (HbA/HbS)
				AA	1		0.72	Sickle cell disease (HbS/HbS)
		**rs33930165**	HbC	CC	137	139	98.56	Normal (HbA/HbA)
				CT	2		1.44	Heterozygous with coding variant – pathogenic for red blood cells (HbA/HbC), protective against malaria
		**rs713040**	β thalassemia & hereditary persistence of fetal haemoglobin (HPFH^{C})	AA	1	140	0.71	Homozygous carrier of a benign variant, no confirmed disease association - variant may be silent or rarely missense, but no functional effect observed
				AG	27		19.29	Heterozygous carrier of a benign variants, no confirmed disease association - variant may be silent or missense, no significant impact on hemoglobin function
				GG	112		80	Homozygous for a benign synonymous variant of the HBB gene, no confirmed pathogenic effect - variant does not alter hemoglobin structure or activity
		**rs72561473**	C>T	CC	133	137	97.08	Normal
				CT	4	137	2.92	Heterozygous carrier of this rare variant, located in the regulatory region upstream of the HBB gene, which could influence its expression and may be potentially associated with beta-thalassemia, although its clinical impact remains uncertain and subject to conflicting interpretations

Supplementary Table S2: Genotypic frequencies and description of the impact of detected chromosome X variants in the Senegalese population

Chr	Genes	RS number	Sexes	Variant information	Genotypes	Observed counts	Total	Frequencies	Consequences/Impacts
X	G6PD	rs1050828	Male	G202A	C	59	59	100	Normal
			Female		CC	57	62	91.94	Normal
					CT	5		8.06	Heterozygous for a coding substitution, associated with a risk of G6PD enzyme deficiency, which can cause chronic non-spherocytic hemolytic anemia; classified as pathogenic or likely pathogenic
		rs1050829	Male	A376G	T	25	34	73.53	Normal
					C	9		26.47	Moderate risk of G6PD deficiency; possible susceptibility to hemolytic anemia under oxidative stress
			Female		TT	18	69	26.09	Normal
					TC	45		65.22	Moderate risk of G6PD deficiency; possible susceptibility to hemolytic anemia under oxidative stress
					CC	6		8.7	Higher risk of G6PD deficiency; symptoms more likely under oxidative stress
		rs5986875	Male	G>A	G	66	66	100	Normal
			Female		GG	63	65	96.92	Normal
					GA	2		3,08	Heterozygote with intronic mutation with no effect on G6PD enzyme activity, and no significant clinical risk of hemolytic anemia
		rs76723693	Male	T968C	A	65	65	100	Normal
			Female		AA	62	65	95.39	Normal
					AG	3		4.61	Rare missense variant, likely pathogenic, associated with G6PD deficiency and increased risk of hemolytic anemia under oxidative stress
		rs917134021	Male	G>C	G	69	69	100	Normal
			Female		GG	63	65	96.92	Normal
					GC	2		3.08	Rare intronic variant of G6PD, likely benign, with uncertain functional effect

* Chr: Chromosomes; **: Information collected from: dbSNP – NCBI[1], ClinVar[2], Osborne et al. (2023), Kariuki et Williams (2020)

[1] https://www.ncbi.nlm.nih.gov/snp/
[2] https://www.ncbi.nlm.nih.gov/clinvar/

Supplementary Table S3: Genetic diversity based on autosomal locus

			H_O	H_E	F_{IS}	F_{ST}
**Zone**						
	Tambacounda		0.05386	0.05379	-0.00388	
	Kedougou		0.05256	0.04779	-0.05160	0
	South Senegal		0.05619	0.06381	0.05165	
**Locus**						
	ACKR1	rs577808287	0.01843	0.01843	~0	0
		rs2814778	0	0	-	-
	Dantu	rs186873296	0.01913	0.01913	~0	0
	HBB	rs334	0.11277	0.11403	0.00223	0
		rs33930165	0.00783	0.00780	-0.00600	0
		rs713040	0.19783	0.20323	0.01333	0.00220
		rs72561473	0.02340	0.02327	-0.00610	0
**Global**			**0.05420**	**0.05513**	**0.00219**	**-**

H_O: Observed heterozygosity; H_E: Expected heterozygosity; F_{IS}: Inbreeding coefficient; F_{ST}: Fixation index.

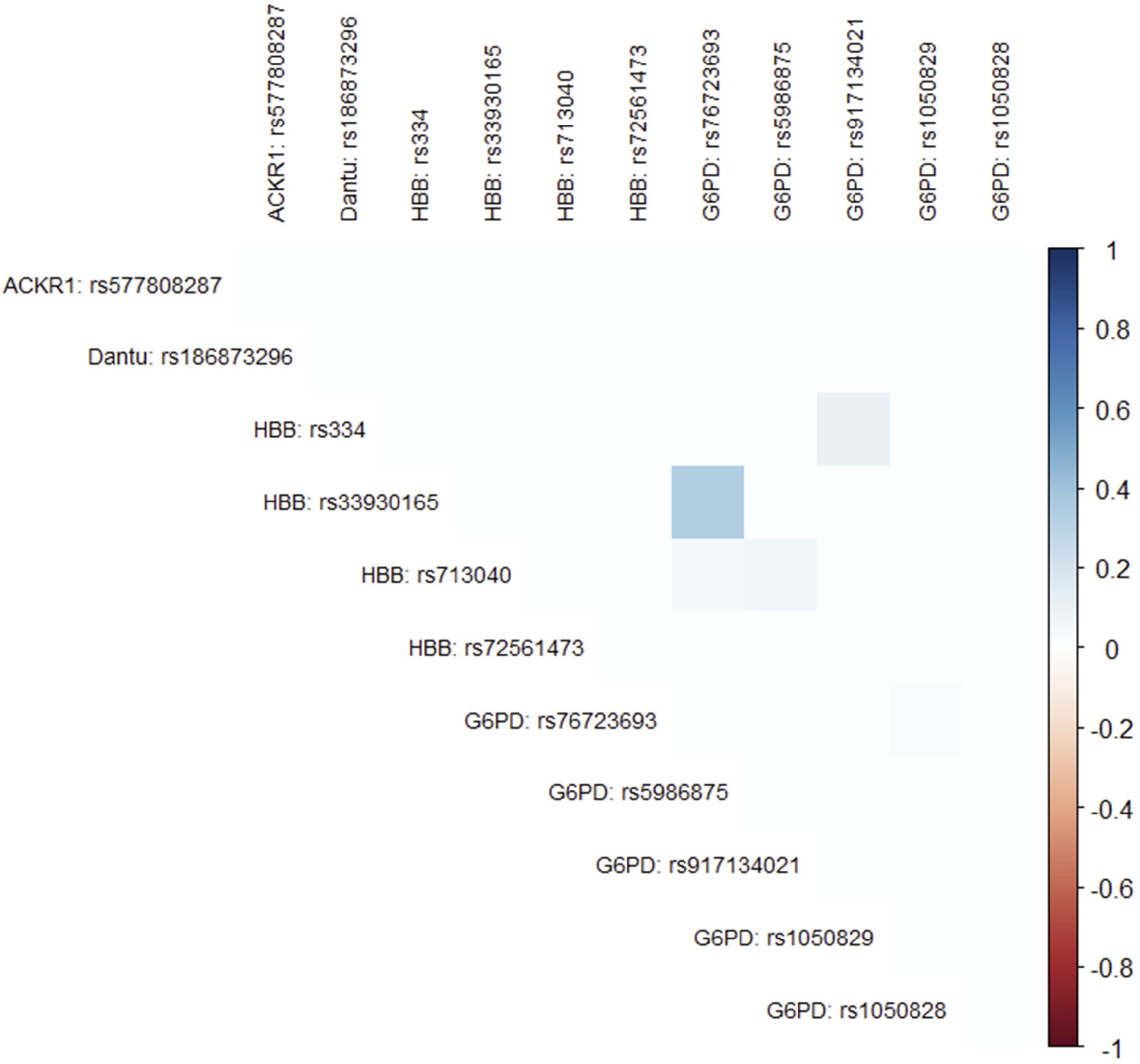

Supplementary Figure S1: Linkage disequilibrium calculated using the R^2 and Fisher's exact test (all p-values are not significant).

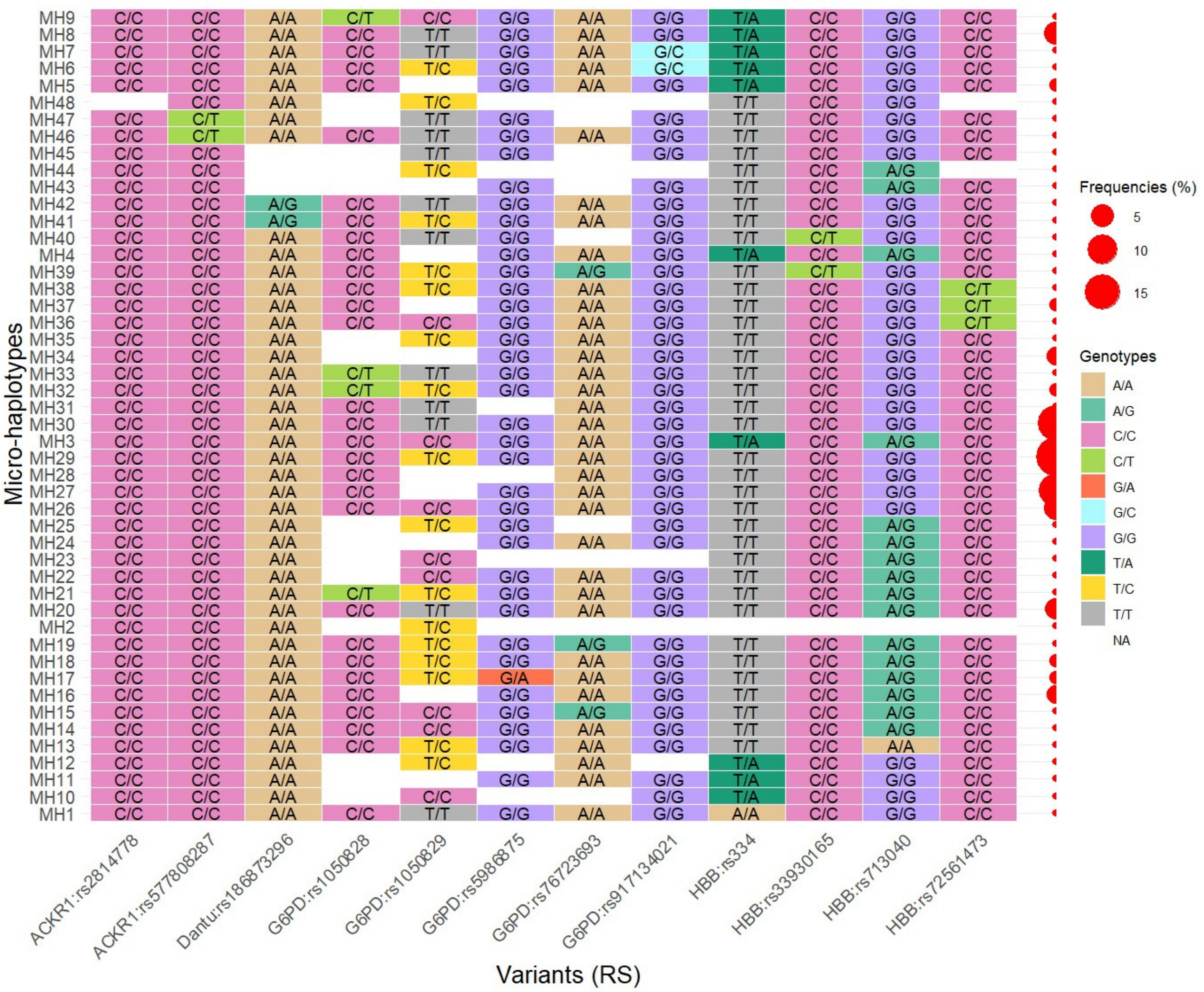

Supplementary Figure S2: Microhaplotype frequencies.
